# Multi-centre deep learning for placenta segmentation in obstetric ultrasound with multi-observer and cross-country generalization

**DOI:** 10.1038/s41598-023-29105-x

**Published:** 2023-02-08

**Authors:** Lisbeth Anita Andreasen, Aasa Feragen, Anders Nymark Christensen, Jonathan Kistrup Thybo, Morten Bo S. Svendsen, Kilian Zepf, Karim Lekadir, Martin Grønnebæk Tolsgaard

**Affiliations:** 1 Copenhagen Academy for Medical Education and Simulation (CAMES) Rigshospitalet, Copenhagen, Denmark; 2 Technical University of Denmark (DTU) Compute, Lyngby, Denmark; 3 Artificial Intelligence in Medicine Lab (BCN-AIM), Universitat de Barcelona, Barcelona, Spain; 4 Department of Fetal Medicine, Copenhagen University Hospital Rigshospitalet, Copenhagen, Denmark

**Keywords:** Health care, Medical research

## Abstract

The placenta is crucial to fetal well-being and it plays a significant role in the pathogenesis of hypertensive pregnancy disorders. Moreover, a timely diagnosis of placenta previa may save lives. Ultrasound is the primary imaging modality in pregnancy, but high-quality imaging depends on the access to equipment and staff, which is not possible in all settings. Convolutional neural networks may help standardize the acquisition of images for fetal diagnostics. Our aim was to develop a deep learning based model for classification and segmentation of the placenta in ultrasound images. We trained a model based on manual annotations of 7,500 ultrasound images to identify and segment the placenta. The model's performance was compared to annotations made by 25 clinicians (experts, trainees, midwives). The overall image classification accuracy was 81%. The average intersection over union score (IoU) reached 0.78. The model’s accuracy was lower than experts’ and trainees’, but it outperformed all clinicians at delineating the placenta, IoU = 0.75 vs 0.69, 0.66, 0.59. The model was cross validated on 100 2nd trimester images from Barcelona, yielding an accuracy of 76%, IoU 0.68. In conclusion, we developed a model for automatic classification and segmentation of the placenta with consistent performance across different patient populations. It may be used for automated detection of placenta previa and enable future deep learning research in placental dysfunction.

## Introduction

Within many fields of medical imaging, methods like convolutional neural networks (CNN) are being used to improve visual diagnostics^1^. In maternal–fetal medicine, CNNs have been developed for recognizing and optimizing standard planes for fetal weight estimation, echocardiography, and neurosonography^2–5^.

One area of fetal ultrasound imaging that remains difficult to evaluate systematically is the placenta, making it an obvious target for neural network analysis. The placenta supplies the fetus with oxygen and nutrients and is the key to several pregnancy complications, including placenta previa, gestational hypertensive disorders, fetal growth restriction, and intrauterine fetal death. Placental volume is the only known morphological factor that can predict adverse events. A small placenta at the first-trimester scan is associated with an increased risk of impaired fetal growth and preeclampsia^6,7^. However, estimating placental volume is time-consuming, and operator dependency is problematic.

For placental volume estimation, semi-automatic 3D placenta segmentation has been successfully performed in previous studies. Yang et al. used a CNN to perform automatic segmentation of placenta, amniotic fluid, and fetus^8^. Looney et al. trained a deep neural network for automated 3D segmentation of the placenta for volume estimation in first trimester placentas^9^. These placentas were segmented automatically using a random walker algorithm after initial seeding by two operators. This was further improved in a recent study by applying a multiclass CNN^10^. Anterior placentas are generally more easily segmented, and classification accuracy improves when the CNN is trained on either anterior or posterior placentas^11^. Studies examining 2D ultrasound placenta segmentation are scarce. Among these, Hu et al. used manual segmentation as ground truth and showed that including a layer containing shadow detection improves performance in images with acoustic shadows^12^.

Most existing studies on placenta classification and segmentation have used structured protocols for image acquisition, which may inflate accuracy at the expense of robustness and generalizability. Few studies have examined how placenta classification and segmentation models perform with different patient populations and datasets as most models are based on single-institution datasets. Finally, there is minimal data on how well these models compare to the performances of clinicians with different levels of ultrasound competence.

To fill this gap, we aimed to develop a model using CNNs to identify the location of placental tissue and perform segmentation of the placenta based on images acquired during routine ultrasound examinations. To examine how well our model performed with different populations, we trained, tested, and validated the model using data from several hospitals across two different European countries, and we determined baseline performance for different groups of clinicians.

## Results

### Results based on the entire test set

#### Image classification

The overall image classification accuracy on the entire test set across trimesters, was 81.42%. Accuracy, precision, recall, and F1 across trimesters are included in Table 1.

**Table 1:** Performance measures and IoU for the model performed on the entire test set across trimesters.

Trimester >	All	First	Second	Third
Accuracy	0.81	0.55	0.84	0.83
Precision	0.88	0.83	0.90	0.82
Recall	0.78	0.43	0.82	0.79
F1	0.83	0.57	0.86	0.80
IoU	0.78 ± 0.15	0.59 ± 0.16	0.79 ± 0.13	0.78 ± 0.15

Classification performance improved with gestational age and was stable from the second trimester. Most mistakes made in the first trimester were due to failure to detect placental tissue as the precision remains stable in the first trimester.

#### Image segmentation

For those test images where placental tissue was detected, the IoU between the prediction and the annotated placenta can be found in Table 1. We note that performance increases from the first trimester and that performance is stable in the second and third trimesters.

### Classification of anterior/posterior placental tissue

**Figure 1:**
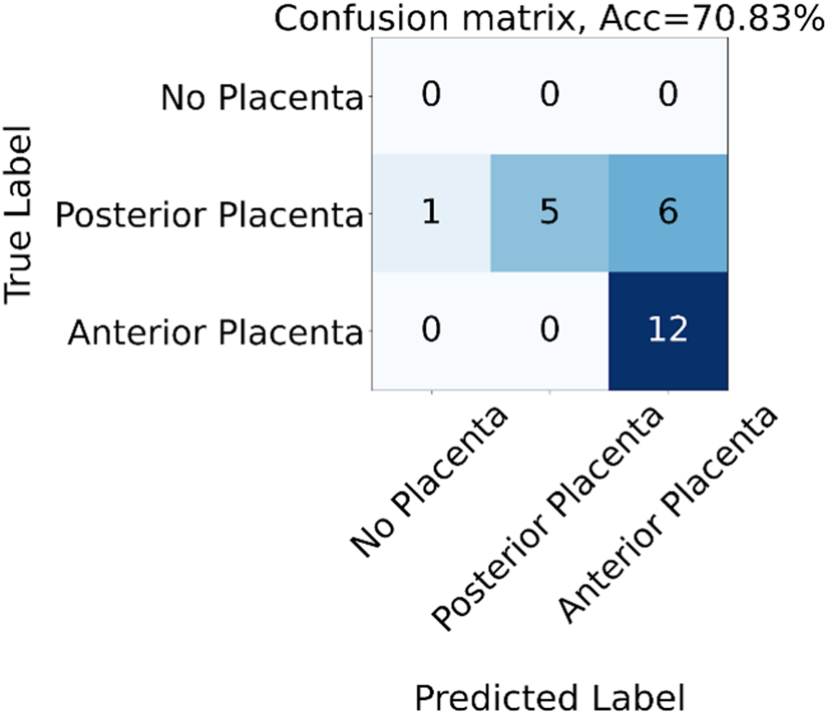
Confusion matrix for classification of anterior/posterior placentas.

The model for distinguishing between anterior and posterior placental tissue yielded an accuracy of 71% (Fig. 1). The model performed better at recognizing the anterior placenta, which is to be expected based on previous research^13^.

### Comparison with different groups of clinicians on a data subset

**Figure 2:**
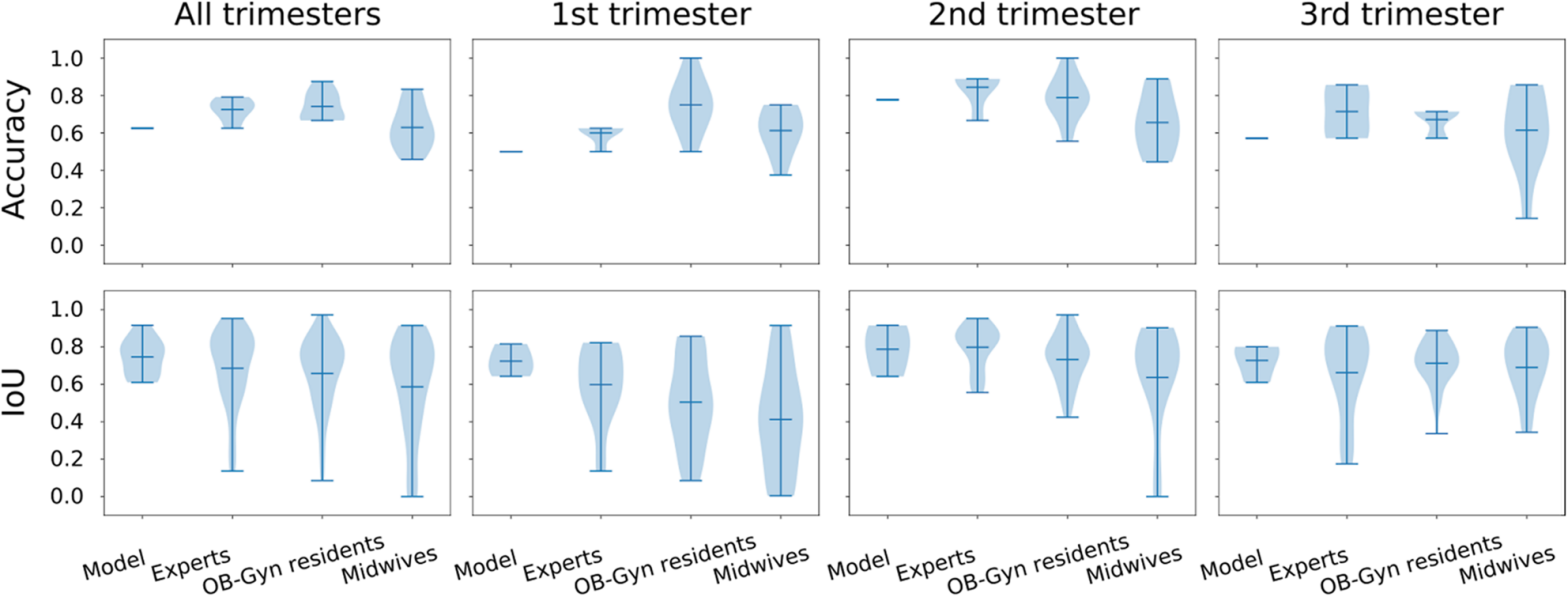
Violin plots of classification accuracy distributions (first row) and distribution of segmentation performance measured by IoU (second row) for different groups across trimesters.

**Figure 3:**
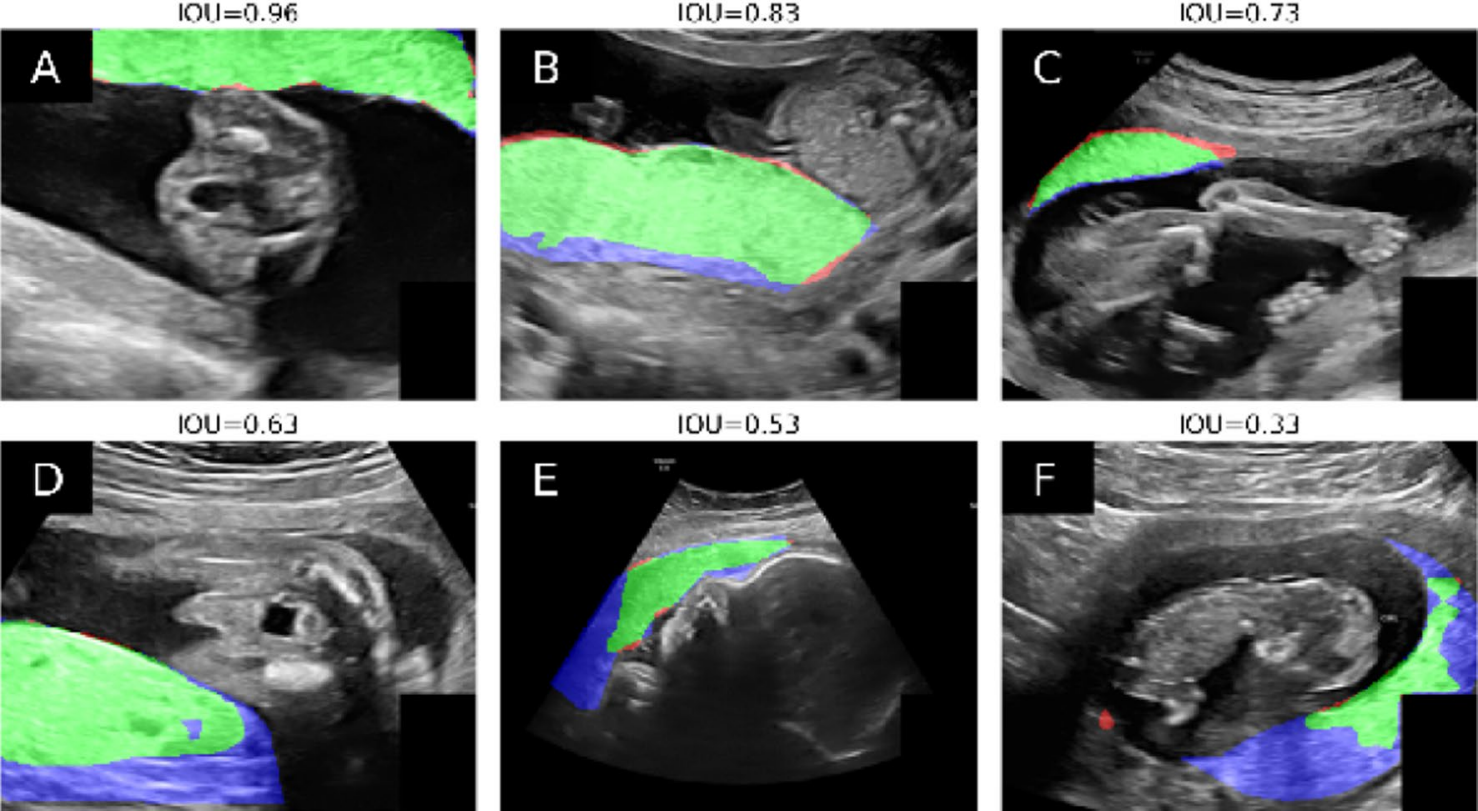
Six annotation examples, including model prediction (red), ground truth (blue) and their overlap (green).

For image classification, where the model detected whether the image contained placental tissue or not, Table 2 compares the model’s accuracy with the performance of different groups of clinicians for a subset of 24 randomly selected ultrasound images. Table 3 demonstrates the segmentation performance of the model and different groups of clinicians on segmentations where both the ground truth and the prediction/clinician indicated that the image contained placental tissue. These comparisons are summarized visually in the violin plots in Fig. 2. Example segmentations along with the ground truth are shown in Fig. 3.

**Table 2:** Classification accuracy on a subset of 24 randomly selected ultrasound images of the model compared to mean and standard deviation of classification accuracy distributions for different groups across trimesters. P-values of t-test are reported in parentheses, using a one-sided, one-sample t-test.

Classification accuracy	All trimesters	1st trimester	2nd trimester	3rd trimester
Model	0.63	0.5	0.78	0.57
Clinicians overall	0.69 ± 0.10 (p = 0.002)	0.67 ± 0.14 (p < 0.001)	0.75 ± 0.15 (p = 0.16)	0.66 ± 0.16 (p = 0.007)
Experts	0.73 ± 0.06 (p = 0.012)	0.60 ± 0.05 (p = 0.0081)	0.84 ± 0.09 (p = 0.10)	0.71 ± 0.13 (p = 0.045)
OB-Gyn residents	0.74 ± 0.07 (p < 0.001)	0.75 ± 0.14 (p < 0.001)	0.79 ± 0.12 (p = 0.39)	0.67 ± 0.07 (p < 0.001)
Midwives	0.63 ± 0.12 (p = 0.46)	0.61 ± 0.12 (p = 0.009)	0.66 ± 0.15 (p = 0.020)	0.61 ± 0.21 (p = 0.28)

**Table 3:** Segmentation performance measured by IoU across trimesters for those subset cases, where images were correctly classified as containing placental tissue by the model or clinician.

Segmentation IoU	All trimesters	1st trimester	2nd trimester	3rd trimester
Model	0.75 ± 0.10	0.72 ± 0.07	0.79 ± 0.11	0.73 ± 0.08
Clinicians overall	0.65 ± 0.22 (p = 0.091, 0.045)	0.51 ± 0.25 (p = 0.071, p = 0.091)	0.73 ± 0.18 (p = 0.28, p = 0.65)	0.69 ± 0.16 (p = 0.36, p = 0.47)
Experts	0.69 ± 0.20 (p = 0.20, p = 0.051)	0.60 ± 0.20 (p = 0.16, p = 0.22)	0.80 ± 0.12 (p = 0.44, p = 0.99)	0.66 ± 0.22 (p = 32, p = 0.19)
OB-Gyn residents	0.66 ± 0.20 (p = 0.09, p = 0.084)	0.50 ± 0.24 (p = 0.068, p = 0.11)	0.73 ± 0.13 (p = 0.25, p = 0.83)	0.71 ± 0.12 (p = 0.42, p = 0.82)
Midwives	0.59 ± 0.26 (p = 0.19, p = 0.011)	0.41 ± 0.28 (p = 0.16, p = 0.13)	0.64 ± 0.27 (p = 0.44, p = 0.35)	0.69 ± 0.16 (p = 0.32, p = 0.40)

The IoU analysis is limited to those cases where the model or clinician correctly classifies the image as containing placental tissue, hence precisely measuring the quality of the delineated placenta in those cases where it is correctly detected. In parentheses are found the p-values resulting from (left) a 2-sample t-test for means and (right) a Levene test for variances.

For the classification problem of detecting whether or not the image contained placental tissue, the model obtained an accuracy comparable to that of the midwives. While this might seem low, the results in Table 3 and Fig. 2 show that *when* the model detects placental tissue, it consistently performs better and more robustly than all groups of experts (Supplementary Information).

### Cross-country generalization

Finally, we evaluated the generalization of the final model on a dataset from another country (Spain) by externally testing it on 100 annotated images from a publicly available dataset from Barcelona. The performance was slightly lower than in the dataset from Copenhagen concerning accuracy but with similar F1 values (Table 4).

**Table 4:** Cross-validation performance on a dataset from Barcelona containing 100 2nd trimester ultrasound images.

Accuracy	0.76
Precision	0.62
Recall	0.62
F1	0.83

For the 25 images where both the prediction and the ground truth detected placental tissue, the IoU was 0.68 ± 0.20.

## Discussion

We developed a model to identify and segment the placenta in ultrasound images. Our model identified the presence of placental tissue with an accuracy of 81%, and in images with correctly identified placental tissue, it reached an average IoU of 0.78.

We benchmarked the model's performance to obstetric staff with different levels of ultrasound experience—fetal medicine experts, obstetrician trainees, and midwives. The model performed at the level of midwives (Table 2) for the classification task, and it consistently yielded better and more robust segmentation of the placenta than all groups of clinicians (Table 3).

Finally, identification of placental location related to the posterior or anterior uterine wall reached an accuracy of 71%.. In alignment with other studies^13^, we found that the model performed better on anterior placentas (Fig. 1).

We have shown that a deep neural network for placenta analysis can perform as well as—and better than—certain groups of clinicians. As such, we have shown that it is possible to remove the most time-consuming part of placenta segmentation—the annotation process. This will facilitate future research on placenta-mediated disorders as it enables efficient analysis of large datasets, thereby making future image analysis of placental disease possible.

This is important as it may help improve prediction of adverse pregnancy outcomes. In particular, hypertensive pregnancy disorders, compromised fetal growth, and fetal death often stem from placental dysfunction—either due to insufficient initial placentation or diminishing placental function with increasing gestational age^14,15^. However,these conditions that compromise both maternal and fetal well-being present themselves without warning, most often too late for preemptive measures. A huge effort has been put into researching predictors of these conditions, yielding some promising but preliminary results. Biomarkers in the maternal bloodstream have been investigated extensively and an association has been found between certain biomarkers and the development of preeclampsia, impaired fetal growth and stillbirth^16–18^. Recently, placental microRNAs coding for genes related to angiogenesis, growth, and immunomodulation have been found to be measurable in maternal blood lending promise to yet another potential source of early detection of placenta-related pathology^19,20^. However, these are limited by small sample size and expensive methodology.

Ultrasound can be used to measure Doppler velocimetry in the uterine artery, which is associated with development of preeclampsia and placental pathology^21,22^, but when it comes to morphological markers of impaired placental function, reliable indicators are scarce. It is well-documented that placental volume is often smaller in hypertensive pregnancies and cases with growth restriction^23,24^. A study by Looney et al. has already demonstrated that it is possible to estimate placental volume in the first trimester using deep learning and that low placental volume by this method is a predictor of low birth weight^9^. However, our results offer interesting perspectives to this. In placental insufficiency and preeclampsia cases, placental tissue often shows histological and macroscopic changes^25^. These structural changes are not visible to the naked eye on ultrasound—and computers may be able to identify such minuscule changes and predict disorders that manifest at a much later point in pregnancy. In fact, Gupta et al. found that AI image texture analysis of the placenta differed significantly between hypertensive and non-hypertensive mothers^26^, and in a much larger study by Hu et al.^12^ authors achieved an accuracy of 81% on the classification of placentas as either normal or abnormal defined by the presence of preeclampsia or fetal growth restriction at birth. Placenta segmentation was achieved using a CNN.

Our findings add to these existing studies and may lead to a novel approach to early prediction of adverse pregnancy outcomes. CNNs alone or in combination with biomarkers may ensure that preventive measures and increased surveillance can be established in time to avoid adverse outcomes. This would revolutionize prenatal care, and our study paves the way to more studies on AI-based prediction of placenta-related disorders using automated segmentation of routine ultrasound images.

Another important future application of our model lies in low-resource settings where the use of ultrasound is limited due to suboptimal access to equipment and qualified personnel. For example, our model can help medical staff identify patients with placenta previa. If this condition is not recognized prior to the onset of labor, both mother and fetus are likely to die from excessive blood loss, as safe delivery is only possible by caesarean section. Our model may enable screening in such settings and save lives. However, studies are needed to determine how best to apply models such as ours for real-time ultrasound imaging using a variety of ultrasound equipment and users with different levels of ultrasound experience.

### Strengths and limitations

Our results are based on a large dataset of 7,500 annotated ultrasound images. Images were obtained as part of routine ultrasound examinations and were highly heterogeneous. A particular challenge was that many images were acquired to present an anatomical structure within the fetus and the placental tissue visualized was often an accidental finding in the image. In other words, most placentas were only visible in a small area of the image. In addition to the inherent complexities of ultrasound images such as acoustic artifacts, image optimization settings, differences in mother and fetus, the examinations were performed by a number of different sonographers using different ultrasound machines.

However, we chose to include all types of images regardless of image quality in our analysis. To achieve higher model performance, more high-quality images dedicated to identifying placental tissue could have been included. However, such training data would have limited generalizability of the model considering real-life’s ‘messy’ data and applications. Consequently, the heterogeneity of our dataset also represents a strength.

Moreover, the large variance in image quality also exposed the fact that placenta identification and segmentation was very difficult for clinicians, including experts, when presented with images of sub-optimal quality. It is a strength that we obtained data on the performance of clinicians with a wide range of clinical experience, which allowed us to better benchmark our model. However, the small number of images in both the cross-validation test set and the dataset presented to the clinicians (24 images) represent a limitation.

Finally, a major strength of our study was the use of cross-validation using a dataset from another country. This cross-validation confirmed that our model was able to perform consistently with different patient populations as well as different groups of clinicians.

## Conclusion

We developed and used CNNs for placenta identification, classification and segmentation. Our model performed slightly worse than experienced clinicians at a classification task but more consistently for the segmentation task than all groups of clinicians.

Our findings have implications for the use of ultrasound diagnostics by untrained health care providers and future research in AI-based placental pathology detection, which may be of paramount importance for predicting a wide range of maternal–fetal placenta-related diseases.

## Methods

### Image annotation

A total of 7,500 ultrasound images were utilized, sampled randomly from obstetric ultrasound examinations recorded in Copenhagen, Denmark from January 1 to December 31 2018. Sample size was based on feasibility. The images were taken as a part of the Danish screening program, including first-trimester aneuploidy screening, second-trimester screening for fetal malformations, and other examinations performed on indication. All ultrasound images are routinely stored locally in the Astraia database (Astraia Software GmbH, Munich, Germany) and we had access to all of these examinations. Finally, images for annotation were anonymized and stored in a secure server.

The presence or absence of a placenta in the images was recorded. Then, for images containing placenta, the annotation tool Labelme^27^ was used to outline the placenta to create the ground truth dataset. This manual annotation was performed by the lead author (LA) for all 7500 ultrasound images.

In addition, in 500 randomly selected images, the placental position was labeled as belonging to either the anterior or posterior uterine wall.

In order to compare model performance to that of clinical staff, five fetal medicine experts, ten OB-gyn residents, and ten midwives annotated 24 randomly selected ultrasound images representing all trimesters (8/9/7 images from first/second/third trimester respectively). The participants gave written informed consent and participation was voluntary. Fetal medicine experts were specialists in fetal medicine, OB-gyn residents were employed in an OB-gyn department but without a specialist degree and midwives were authorized midwives without prior ultrasound experience. There was no subject overlap between this additional test set and the training set.

### Ethics

This study was approved by The Danish Data Protection Agency (protocol no P-2019-310) and The Danish Patient Safety Authority (protocol no 3-3031-2915/1). Under Danish law The Danish Patient Safety Authority can give permission to use data from patient charts for research purposes without consent from the individual patient. This was done prior to data acquisition.

This study has been conducted in accordance with the relevant guidelines and regulations.

### Model architecture and training

The Mask R-CNN model proposed by He et al.^28^ was used in this study due to its wide use in instance segmentation in many different domains. The model was initialized with a backbone pre-trained on the ImageNet database and trained on our data using PyTorch^29^. During training, the model was supplied with images and corresponding binary masks, encoding the location of each region of placental tissue in the image on a per-pixel basis. The masks were extracted from the polygons created during the annotation process. During inference, the model located all regions predicted to contain placental tissue and outputted a mask for each region. In addition, the model predicted each predicted confidence score between 0 and 1 for the presence of placental tissue.

### Images and partitions

Out of the 7500 images that were screened, 2130 images were found to contain placental regions during the annotation process. These were randomly split into a training set, a validation set, and a test set containing 1704, 208 and 218 images. In addition, 208 and 218 images with no placental regions were added to the validation- and test-sets to estimate the robustness of the model to false negatives. The training/validation/test partitions were created to ensure that no subject’s images would appear in more than one partition.

Of the 500 images screened and annotated for anterior or posterior placental position, 164 images showed placental tissue. These were divided into training-, validation- and test-partitions containing 116, 24 and 24 images respectively. The validation- and test-sets were made with an equal number of anterior and posterior placental regions. No images without placental regions were added in this experiment.

The validation sets were used for finding optimal hyperparameters, including the acceptance threshold, training duration, and the amount of Random Augmentation to be applied.

An additional test was performed on 100 annotated images from a publicly available dataset from Barcelona. These images were acquired using Voluson E6, Voluson S8 and Voluson S10 (GE Medical Systems, Zipf, Austria), and Aloka (Aloka CO., LTD), while the Danish dataset was recorded on Voluson E6, Voluson S8 and Voluson S10 (GE Medical Systems, Zipf, Austria).

### Random augmentation of training data

The model was trained both with and without the addition of Random Augmentation. The Random Augmentation was added to the training data to make the model more robust. Geometry-transforming augmentations such as shearing and rotation were added to both the images and corresponding masks, whereas per-pixel augmentations such as brightness or color inversions were only applied to the images. The amount of augmentation applied constitutes a hyperparameter adjusted by tuning the number of random augmentations applied to each image.

For each image in the training partition, a number of randomly augmented images were created and added to the set. The non-augmented version was kept in the training set as well.

### Training

The model was trained on the training set for 100 epochs using the Adam optimization algorithm. The model performance was computed on the validation set after each epoch, and the best-performing model was saved as the final output model. The model was trained on an Nvidia Quadro RTX 6000 24 GB GPU with a batch size of 5 and a training time of approximately 6 min per epoch.

### Model prediction processing

During inference, the output of the model was processed in two steps:

#### Confidence

All predicted placental regions with a confidence score below a set threshold (the acceptance threshold = 0.5) were discarded.

#### Overlapping predictions

If multiple placental regions were predicted by the model in an image and one of the regions was located entirely inside another region, the region with the lowest confidence was discarded.

### Evaluation

The model was evaluated on the validation- and test-sets using two different approaches:

#### Image classification

The model was evaluated on its ability to detect whether there was placental tissue in an image or not, without considering the correspondence between the predicted placental regions and the ground truth regions. We approached the task as a binary image classification problem, in which a segmentation map, generated by either the model or an annotator for a given image, is mapped as *empty* or *non-empty*.

In addition to quantifying the model's performance concerning the ground truth annotation, we compared the model’s accuracy with human performance.

#### Image segmentation

Next, the model was evaluated on its ability to locate the placental regions accurately. The overlap between the annotation and the prediction was quantified as the Intersection Over Union fraction (IoU).

Next, we compared the model performance with the performance of the three groups of clinicians with different levels of ultrasound experience (5 fetal medicine experts, 10 OB-GYN residents, and 10 midwives) on the 24 annotated test images; we compared the IoUs of the model with the ground truth to the IoUs of the annotators with the ground truth. For the IoU comparisons, we only included images containing placenta in our analyses.

All analyses were further split across the three trimesters.

#### Cross-country generalization

To determine how well the final model performed with data from a different population, we used an open-access dataset^5^ from Barcelona, Spain, as the final cross-validation test. We selected 100 s-trimester ultrasound images and manually annotated these images using the same annotation procedure as described above. Model performance was then compared across datasets.

### Reporting

The results are reported in accordance with the TRIPOD guidelines^30^.

## Supplementary Information

Supplementary Information.

## Data Availability

Data are not publicly available due to Danish data protection regulations. The framework created during the study can be made available on reasonable request by contacting the corresponding author.
